# Crick’s sequence hypothesis - a review

**DOI:** 10.1080/19420889.2019.1594501

**Published:** 2019-03-24

**Authors:** Keith Baverstock

**Affiliations:** Department of Environmental and Biological Sciences, University of Eastern Finland, Kuopio Campus, Kuopio, Finland

**Keywords:** Peptide folding, misfolded proteins, mutation, GWAS, moonlighting proteins, maximum entropy, chaperones: native state

## Abstract

Health care based on gene sequencing and genomics is increasingly becoming a reality: it is timely to review Crick’s sequence hypothesis for its fitness for this purpose. The sequence hypothesis is central to the prediction and correction of disease traits from gene sequence information. Considerable success in this respect has been achieved for rare diseases, but for the dominant part of the human disease burden, common diseases, little progress has been made since the completion of the sequencing of the human genome. It is argued here that the sequence hypothesis, namely the assumption that peptides will fold spontaneously to the native state protein, thus retaining the information coded in the originating genes, is not supported by a realistic physics-based assessment of the peptide to protein folding process.

## Introduction

The sequence hypothesis lies at the very nexus of the genomic enterprise to improve human health, the crowning achievement of which was the sequencing of the human genome, completed in 2003. In the UK, a company, “Genomics England”, collaborates with the National Health Service to develop a strategy for the future of health care based on exploiting DNA sequence data to improve diagnosis and treatment. To date 100,000 volunteered genomes have been sequenced. The aim is somewhat modest, focussing on rare inherited diseases (and personalised medicine for cancers). According to Genomics England one in 17 people are born with a rare inherited disease and they are generally believed to account for some 15% of the disease burden. However, associating rare disease traits with specific genes was happening well before 2003. For example, a form of the rare heritable Ehlers-Danlos syndrome was first linked to a gene in 1972 [1]. In a recent evaluation of the role of molecular genetics since 2003, Paneth and Vermund conclude that despite the laudable goals and the investment of some 10 billion USD in the USA alone, molecular genetics has yet to contribute to measurable public health advances [2].

In terms of relating a disease trait to abnormal alleles in the DNA sequence, the success that has been evident in the case of rare diseases for some considerable time is not matched for common diseases. In the last 15 or so years, with the benefit of the human genome sequence, several common disease traits have been studied in large groups of patients using genome wide association studies (GWAS). Typically, a small fraction of the perceived inherited risk has been attributed to abnormalities at several gene loci, each with a very small effect. The remainder of the inherited risk has yet to be found [3]. For example, some 3.4% of the heritable risk of schizophrenia, assessed in some 37,000 patients, has implicated 108 genetic loci [4]. Common diseases are consequently termed polygenic. Striving to improve the statistics by increasing patient numbers tends to increase the attributable percentage of risk, but also the number of gene loci involved, sometimes very significantly. It has been suggested that the term 'omnigenic' may be more appropriate than polygenic [5]. Clearly, there is little utility as far as diagnosis and treatment is concerned in knowing that hundreds of genes, each with miniscule effect, are responsible for a trait [6].

However, the possibility exists that common diseases are not, in fact, genetic in terms of their causation, i.e., they are not caused by gene mutations and, therefore, there is no relationship to be found between abnormal alleles and common disease. Here I review the evidence relating to the sequence hypothesis to assess the fitness of molecular genetics to play a leading role in health care.

## A paradox

First consider this interesting paradox. The adult human is a composite organism consisting of about 99% by mass of eukaryotic cells and about 1% of bacterial cells, although in terms of cell number, bacterial cells dominate. The eukaryotic cells harbour some 20,000 protein coding genes and the bacteria, collectively among many species, some 9 million genes in the human gastro-intestinal tract alone [7]. It seems, if genes are indeed the basis for phenotypic traits, paradoxical that the eukaryotic component of the organism, which has by far the bigger role in forming the human phenotype, has so many fewer genes than the microbiome. Of course, selective transcription of eukaryotic gene sequences carrying several exons and the editing of mRNA, pushes up the number the proteins those 20,000 gene sequences can produce. One specific gene, found in fruit flies can, in theory, produce ~38,000 distinct mRNA molecules and thus, proteins [8]. Admittedly, this is a rare example. Prior to the sequencing of the human genome it was estimated that the eukaryotic cells of the human produced in the region of 150,000 proteins, well short of the 9 million proteins produced by the bacteria in the human gastro-intestinal tract. Is there another factor that could amplify the number of active proteins in eukaryotic cells?

## The origin of the sequence hypothesis

In 1958, five years after the publication of the structure of DNA, Francis Crick published his thoughts on how the information in the base sequence of DNA was translated into information in the phenotype. He proposed the ‘sequence hypothesis’ and the ‘central dogma’ [9]. The information flow was, he proposed, one way only from the DNA through RNA to protein. Interestingly, he did not mention the intervening peptide. In 1970, in a paper reflecting on the 1958 paper, he says of the folding of the peptide to the protein:

‘Because it was abundantly clear by that time [1958] that protein had a well-defined three-dimensional structure, and that its activity depended crucially on this structure, it was necessary to put the folding process to one side, and postulate that by and large the polypeptide chain folded itself up.’ [10].

This is essentially the sequence hypothesis: the folding of the peptide *spontaneously* to the native state entails the retention, in the protein, of the information in the DNA sequence. There is, therefore, no deep theoretical foundation for the sequence hypothesis, it was, at its origin, pure expediency. However, the American biologist, Christian Anfinsen, showed in 1961 that if denatured (converted from protein to peptide) in dilute aqueous solution in a test tube, ribonuclease would fold itself back to the active protein when the denaturing conditions were removed. However, the refolding process was very slow, and Anfinsen recognized that the process was much faster in the living cellular environment. In his Nobel lecture he says:

‘It is certain that major advances in the understanding of cellular organization, and of the causes and control of abnormalities in such organization, will occur when we can predict, in advance, the three-dimensional, phenotypic consequences of a genetic message.’ [11]

This latter objective, of a general method to predict protein structures from peptide sequences, has yet to be realized [12] and indeed it can be argued, on the grounds of basic physics that it will never be realised [13]. This is because the environment in which folding takes place influences the folding.

## The peptide to protein folding process

Anfinsen’s conclusion, from his study of the kinetics of ribonuclease refolding in the test tube, was that it was a trial and error process [14]. Other folded versions than the lowest energy state, the native form, were ‘visited’ in the folding process and had to unfold and re-fold, perhaps many times before reaching the native state. To minimize aggregation between peptides in the folding process Anfinsen had carried out his experiments in very dilute aqueous solution. This is an ‘ideal’ situation, since the cellular cytoplasm is molecularly very crowded, up to 30% of the volume being dissolved molecules [15], yet aggregation does not seem to be a major problem and folding can take place orders of magnitude faster than in Anfinsen’s experiment.

In 1988 it was discovered that the cell solves the aggregation problem using chaperone proteins [16]. The term was coined in 1987 by John Ellis [17]. Proteins that cross the mitochondrial membrane need to be in the peptide state which is stabilised outside the membrane by the heat shock protein HSP 70. After crossing the membrane, the peptide refolds to the protein in association with HSP60 [16]. Chaperones are not thought to actively fold the peptide, but rather provide an environment in which the folding can take place without the interference of other molecules. HSP 60 was found to be homologous with a protein complex GroEL, which functions in bacteria in association with GroES [18]. Structurally, GroEL is a hollow ‘barrel’ like molecule open at one end, which can be closed by a GroES molecule. The peptide is said to enter the cavity, which is then briefly closed, and then rapidly emerges in the folded state [19]. The first question that arises is “what folded state?”. As there can be many, some of energy only slightly above that of the native state [20], why should the emerging structure be the native structure? If the chaperone provides only the environment that minimizes aggregation, it does not solve the problem of the time taken to fold.

Chakrabarty et al have proposed a peptide folding model whereby GroEL hydrolyses ATP residues to push a misfolded protein back towards the peptide state and then releases it [21]. After release the peptide can find another GroEL cage to attempt folding again. The stimulated denaturation of misfolded proteins when conducted in the test tube speeds the folding process, but not by enough to give parity with folding in the cell cytoplasm.

Protein folding is a *natural* process underpinned by the 2^nd^ law of thermodynamics and the principle of least action [13] in which the entropy of the folding peptide increases at the expense of its free energy. In the test tube (compared to the cell) the aim of the process is to produce the native state of the protein, but as interactions across different parts of the randomly coiled peptide develop, they lead to a variety of structures that are “cul-de-sacs” in terms of the route to the native structure. They are, in the context of that structure, misfolded, but their minimum energy can be close to that of the native state and the energy barriers to be crossed to unfold them are high, so they have a degree of stability [20].

This is probably also true in the cytoplasm, however, there is an interesting consequence in the cell. Firstly, since a given chaperone can assist the folding of several different peptides it can be assumed that the native state protein cannot be detected as such by the chaperone. Thus, chaperones will release into the cytoplasm “misfolded” proteins more often than native state proteins because there are many more such “misfolded” structures. These will be in addition to partially denatured proteins that the chaperone has denatured. It is known that the mammalian cell contains a significant proportion of partially denatured proteins [22] which are active, despite their denatured state and in some cases adopt an active state with respect to a given binding site as they approach it [23]. Furthermore, most transcription factors contain intrinsically disordered domains, the functionalities of which are context dependent [24]. The cytoplasm of the cell does not, therefore, exist in the highly ordered state envisaged, for example, by the genetic regulatory network (GRN) hypothesis [25] with enzymes acting as very specific ‘keys’ to specific ‘locks’, as enzymic action was originally envisaged by Emil Fischer in 1890.

Fonin et al show that the influence of the crowded environment of the cell on the folding process of the significant fraction of intrinsically disordered proteins can be very complex. Depending on the identity of the peptide, crowding can accelerate folding or favour unfolding [15]. It, therefore, seems probable that misfolded proteins will, at any given time, be present in greater numbers than native state proteins. This raises the question: “have these proteins (with variant active sites) been exploited during the process of evolution and, thus, have been enabled to carryout important functions in their own right?” If so, the complement of proteins in the cytoplasm of eukaryotic cells is considerably, but incalculably, increased by misfolded and partially folded versions of proteins, carrying relevant information that contributes to the phenotype, but is not related in any way to that encoded in the gene sequence from which they are derived.

## Discussion and conclusions

From the evidence we have, the logical conclusion is that the cellular cytoplasm is populated by small proteins that have folded or misfolded themselves, proteins that have folded or misfolded in association with chaperones, proteins that have been folded, and partially denatured proteins and random coil peptides. Order emerges out of this melange as various proteins become activated and contribute their information to the emergence of the cellular phenotype by interacting with one another. In the conventional paradigm these interactions are exclusively of native state proteins that carry the information encoded in the base sequences of genes. If it is assumed (and this is a guess just to illustrate the situation) that there are on average 10 misfolded proteins for every native state protein, then at least 90% of the protein content of the cytoplasm is inactive in terms of the cellular phenotype. If, on the other hand, the misfolded proteins are active (providing information) in the emergence of the phenotype, then they are carrying information that is *unrelated* to that in the gene sequence from which they were derived. This raises the questions: what is the information they are carrying and where does it come from?

Before trying to answer those two questions, it is useful to ask if it is a realistic possibility that misfolded proteins are active in producing the phenotype. That they are there cannot be in doubt if Anfinsen’s conclusion that peptide folding is a trial and error process [14] is correct. His conclusion is not controversial and seems to be the most likely, or indeed only, explanation for the slowness of the peptide folding process *in vitro*. If they were present and totally inactive, they would act to inhibit the functions of the cell, if in no other way, sterically. However, if active, they would serve to increase considerably the number of proteins active in eukaryotic cells.

The problem is that if they are active it is not possible to detect them, or know what they do, or what their folded structure is. The reason for this is that if isolated from the cell so that their structure can be determined under test tube crystallising conditions, they would adopt the native, or “correctly folded” structure associated with the peptide from which they were derived. They also, of course, have a transient existence in the test tube during the crystallisation process, but they cannot, with present technology, be readily isolated for structure determination. Furthermore, they cannot be detected by current protein separation processes as these measurements are carried out on denatured proteins. They are what Robert Laughlin calls “dark corollaries” [26] – unknowable, at least for the present, but perhaps essential features of the operation of the cell.

This conclusion is highly relevant to the validity of the conventional molecular genetic paradigm: it means that the information in the gene sequence is likely a small fraction of that which contributes to the phenotype: if only 1 in10 proteins in the cytoplasm exists in the native state at any given time, 10%. Since this 10% figure is necessarily a guess and it could be smaller, it is tempting to think that this phenomenon might be the reason why only a small fraction of the assumed inherited genetic component has been accounted for in terms of abnormal alleles in common diseases. However, GWAS do not correlate traits with specific genes, rather they infer the involvement of specific genes in traits, based on the abnormalities detected in the gene sequence of the patient: that gene sequence is only associated with the protein by dint of the sequence hypothesis. If the protein that is acting to produce the phenotype is not in the native state, there is no causal connection between the gene sequence and the phenotype.

This last statement may appear to be at odds with the fact that there has been considerable success in understanding rare diseases in terms of specific anomalies in specific genes. Rare traits overwhelmingly entail a single gene and, therefore, a single protein. An association between rare traits and specific genetic anomalies does not, however, necessarily imply that the proteins involved have adopted the native state. It only implies that the protein with the anomalous structure is not active. Rare diseases are akin to Mendel’s flower colour trait, where a mutated transcription factor is unable to trigger the production of anthocyanin in pea plants [27]. The very rarity of traits that can be definitively attributed to mutational damage supports the contention that effective mutations are rare and that the cause of common diseases is not, therefore, mutational damage,

What is the origin of information in the chaotic melange of peptides and proteins in the cellular cytoplasm described by Fonin et al [15]? The peptide folding process is dissipative, thus, according to the 2^nd^ law of thermodynamics, generates entropy. This entropy can take the form of information, so the origin of the information, which takes the form of molecular structure, is not necessarily a problem. The outstanding question is why information generated that way would be relevant to the cellular phenotype. Clearly, in the context of a paradigm proposing upward transmission of information from the gene to the phenotype, misfolded proteins cannot have a role. However, if causality runs in the other direction, from the phenotype downwards, then misfolded proteins could have found a role over the course of evolution, just as it is assumed that new variation (novel proteins) arising from random mutation is assumed to have found a role: in effect, misfolded proteins “offer” the phenotype opportunities to make use of their information. A model for biology based on such downward causation and based on thermodynamics, has been proposed [28].

If misfolded proteins carried out a role in cellular biology, we would expect to find multiple roles for a single peptide sequence. This has, indeed, been found in the context of “moonlighting proteins”: that is, proteins that perform more than one role in an organism [29,30]. There are several examples of enzymes active in S. *cerevisiae* metabolism that perform one or more other functions in the same species. The phenomenon is found in other yeast species and has also been observed in other eukaryotic cells, although it has been more thoroughly studied in S. *cerevisiae*. Discovery has usually been by chance and so far, predicting moonlighting has proved elusive.

In conclusion, there is a strong empirical case to be made for Crick’s expediency-based sequence hypothesis being at least unproven, but most likely invalid. Yet it crucially underpins molecular genetics, which has been, for biology, unprecedently funded. Since the late 1980s, among other programmes, the National Human Genome Research Institute at the National Institutes of Health in the USA has consumed 10 billion USD [2]. If there is no contiguous information flow from the genotype to the phenotype there is no rationale for GWAS, the methodology that has dominated the relevant literature in the past decade: such studies are mostly measuring either noise or some other feature of the study population. Although rare disease traits are attributable to mutations in specific genes, it seems that generalising this association to traits described as polygenic or omnigenic is not justified. Since these traits comprise some 85% of the disease burden, the genomic approach to health care cannot be comprehensive. It is, therefore, a matter of public health concern that so much research effort and resource is devoted to GWAS of increasingly large populations of patients (tens of thousands) with common diseases to improve the statistical power of studies that, even if they were not already practically valueless in terms of their utility, are likely not measuring anything meaningful at all.

## Notes

1. This is another way of viewing Anfinsen’s experiment with ribonuclease. He observed the ribonuclease peptide progressing from the state of maximum free energy towards the state of maximum entropy, the stationary, in the context of the folding process, state, or native state structurally, at which point the ribonuclease activity (information) appeared. Other activities (information) may have appeared at the local maximum entropies of many misfolded peptides, but had he been able to detect them, they would have been transitory in his experiment. This perspective generalises, what at first sight appears to be, a ‘specific case’ study testing Crick’s sequence hypothesis, although he makes no reference to Crick.

2.(https://www.biorxiv.org/content/early/2018/12/04/485441).
